# PSYCHOLOGICAL MECHANISMS THAT UNDERLIE GOAL EXPECTANCY AMONG RETIREES

**DOI:** 10.1093/geroni/igz038.466

**Published:** 2019-11-08

**Authors:** Cindy Tsotsoros, Anna Mooney, Joanne Earl, Douglas Hershey

**Affiliations:** 1 Oklahoma State University, Stillwater, Oklahoma, United States; 2 Macquarie University, Sydney, Australia, Australia

## Abstract

The way individuals envision, formulate, and strive to meet retirement goals is poorly understood. In particular, few studies have focused on the goal setting process of individuals who have already retired. In this investigation, the authors replicate and extend Hershey and Jacobs-Lawson’s (2009) model of retirement goal expectancy across five retirement resource domains. Their (path) model posits individuals’ perceptions of the consequence of failing to achieve a goal determines the perceived importance of the goal. Perceived goal importance, in turn, determines the effort individuals allocate toward achieving the goal (goal striving). And goal striving, in turn, predicts the perceived likelihood the goal will be achieved (goal expectancy). This basic model was empirically tested across five key retirement resource domains (health, physical, social, cognitive and emotional) identified by Wang and Shi (2013). The sample of 698 American retirees (Mage=77.14 years, SD=6.00) was divided into four subgroups: males and females, aged 66-77 and 78-94. Twenty theoretically-driven path analysis models were tested using AMOS (i.e., four subgroup models across five retirement domains). Differences were observed across subgroups and domains in terms of the magnitude of path coefficients and the amount of accounted variance in goal expectancy criterion measures (R2 values ranged from .26-.67). Overall, the path model was effective at capturing variability in retirement goal expectancy. Findings not only provide a synthesis of the 2009 goal model with Wang and Shi’s Dynamic Resource Theory, but they also suggest areas in which retirement intervention specialists can intercede to increase the likelihood of goal attainment.

